# The Influence of Diet Change and Oral Metformin on Blood Glucose Regulation and the Fecal Microbiota of Healthy Horses

**DOI:** 10.3390/ani11040976

**Published:** 2021-04-01

**Authors:** Aaron C. Ericsson, Philip J. Johnson, Lyndsy M. Gieche, Chelsea Zobrist, Katie Bucy, Kile S. Townsend, Lynn M. Martin, Alison M. LaCarrubba

**Affiliations:** 1 University of Missouri Metagenomics Center, Department of Veterinary Pathobiology, College of Veterinary Medicine, University of Missouri, Columbia, MO 65201, USA; 2 Department of Veterinary Medicine and Surgery, College of Veterinary Medicine, University of Missouri, Columbia, MO 65211, USA; katie.bucy1@gmail.com (K.B.); townsendks@missouri.edu (K.S.T.); martinlyn@missouri.edu (L.M.M.); lacarrubbaa@missouri.edu (A.M.L.); 3 College of Veterinary Medicine, University of Missouri, Columbia, MO 65211, USA; lgiechedvm@gmail.com (L.M.G.); chelsea.zobrist@outlook.com (C.Z.)

**Keywords:** equine, metabolic syndrome, glucose tolerance

## Abstract

**Simple Summary:**

Horses are susceptible to a condition known as Equine Metabolic Syndrome (EMS), which is somewhat similar to metabolic syndrome and type II diabetes in people, and is characterized by elevated insulin levels and increased susceptibility to other adverse health outcomes. Common treatments, including change to an all-hay diet and a drug called metformin, may provide benefits through modulation of intestinal bacteria, collectively known as the gut microbiota. In the studies reported here, horses undergoing such a diet change, regardless of the presence of metformin, showed a significant expansion in their fecal microbiota of a specific, poorly characterized group of bacteria. Notably, this phylum is distantly related to the bacteria found to expand in the fecal microbiota of mice and humans following metformin administration, suggesting removal from pasture to reduce the caloric intake may exert effects on the equine microbiota similar to those seen in other host species treated with metformin.

**Abstract:**

Common treatments for Equine Metabolic Syndrome (EMS) and associated conditions include removal from pasture and adoption of an all-hay diet. Pharmacological treatments for EMS include metformin, a biguanide antihyperglycemic agent also administered to people to help improve glucose tolerance and insulin sensitivity. Both treatments may work, at least partially, through the gut microbiota, yet little is known regarding these effects in the equine host. To determine the influence on the fecal microbiota of this diet change and administration of metformin, six healthy horses were removed from pasture and switched to an all-hay diet, with four of those horses also receiving oral metformin for seven days. Control horses (*n* = 24) remaining on pasture and receiving no metformin were sampled at the beginning and end of one week. All samples were subjected to 16S rRNA sequencing, and horses undergoing the diet change were subjected to an oral sugar test twice, one week apart. Characteristic changes in the microbiota following diet change included the significant expansion of the phylum Kiritimatiellaeota. As Kiritimatiellaeota are related to Verrucomicrobia, found to expand in the microbiota of mice and humans in response to metformin, this taxon may represent the cognate microbes in equine hosts.

## 1. Introduction

Equine Metabolic Syndrome (EMS) represents a constellation of risk factors for endocrinopathic laminitis in horses, centrally associated with insulin dysregulation (ID) and frequently, but not always, accompanied by obesity [1,2]. ID can manifest as basal hyperinsulinemia, an excessive or prolonged hyperinsulinemic response to oral or IV carbohydrate challenge, and/or tissue-level insulin resistance. Reaching epidemic proportions, hyperinsulinemia has been reported in roughly one in five healthy, nonlaminitic horses in the U.S. [3,4] and 27% of ostensibly healthy ponies in an Australian study [5]. The primary clinical sequela of EMS is endocrinopathic laminitis, while other effects on the cardiovascular system and fertility have been suggested [6]. Hyperinsulinemia should thus be considered an indication for preventive measures intended to reduce plasma insulin levels, increase tissue insulin sensitivity, and lessen the risk of laminitis.

Just as in people, lifestyle changes are the cornerstone of these preventive and therapeutic measures, including increased physical activity, obesity reversal, and dietary restriction, often in the form of limiting or completely eliminating access to pasture and concentrates. Rather, feeding hays with low starch and sugar content is a standard component of therapeutic management for EMS, and the recent European College of Equine Internal Medicine (ECEIM) consensus statement on EMS recommends a daily allowance of 1.25% to 1.5% actual body mass (BM) as dry matter intake, typically corresponding to a digestible intake of 64% to 94% of maintenance requirements [7].

Pharmacological interventions often include the drug metformin, marketed under many different trade names. Metformin, a biguanide antihyperglycemic, appears to exert multimodal effects in people including decreased hepatic gluconeogenesis and increased tissue insulin sensitivity, along with possible anorexiant effects. Despite being the most commonly prescribed oral medication for type II diabetes in humans however, complete understanding of the mechanisms of action of metformin is still unclear and likely multimodal. Several lines of evidence suggest that metformin activates AMP-activated protein kinase (AMPK), and due to its low lipophilicity, enters target cells directly via the SLC22A1 transporter [8,9]. Other studies however, in animal models and human cohorts, provide evidence of a gut microbiota (GM)-mediated mechanism of action [10,11,12,13]. Specifically, multiple prospective studies performed in rodent models, and epidemiological studies of human patients, have identified *Akkermansia* spp., a genus of commensal bacteria within the phylum Verrucomicrobia, as potential mediators of the clinical effects of metformin, and candidate markers of appropriate glucose regulation and metabolic health [11,14,15]. Notably, Verrucomicrobia is a dominant phylum within the horse hindgut, contributing roughly 10% of the bacterial DNA found in the left dorsal colon of nine healthy horses in one survey, and a substantial colonizer of the mucosa in all regions of the equine gastrointestinal tract [16]. Counter-intuitively, increased abundance of Verrucomicrobia has been identified as a characteristic feature of horses with EMS [17], suggesting this phylum might serve as a marker of poor glucose tolerance and insulin regulation in horses, in contrast to its relationship with those physiological features in pre-diabetic humans and mouse models.

While some studies have failed to detect an effect of oral metformin on insulin sensitivity in insulin-resistant (IR) ponies [18] and obese mares [19], Durham et al. reported beneficial effects of metformin on insulin sensitivity and pancreatic β-cell function in IR ponies [20]. Rendle et al. showed significant metformin-induced reductions in peak glucose, area under the curve, and insulin concentration in healthy horses subjected to an oral dextrose challenge, and similar metformin-associated improvements were seen in glucose tolerance and insulin sensitivity following IR induced by dexamethasone [21]. Moreover, the poor bioavailability of metformin in horses [22,23] makes a GM-mediated or other local effect of metformin more likely.

Considering the links described above between metformin and its effects on the GM and glucose regulation, we hypothesized that the common treatments for EMS, i.e., change from pasture to an all-hay diet and administration of metformin, might act through GM-mediated mechanisms. The primary objective of this study was therefore to evaluate the changes occurring in the fecal microbiome of horses moved from pasture to an all-hay diet and receiving metformin. A second objective was to determine the effect of metformin administration on glucose tolerance, and identify associations (if present) between any changes in glucose tolerance and the microbiota. To achieve these objectives, healthy adult teaching horses were removed from pasture and placed on an all-hay diet, with a portion of those horses also receiving oral metformin. Fecal samples were collected daily beginning immediately upon removal from pasture and compared to control horses remaining on pasture. Regulation of blood glucose was assessed via oral sugar tests (OST) before and the day after discontinuation of metformin treatment.

## 2. Methods

### 2.1. Horses and Experimental Design

All horses removed from pasture and/or administered metformin were part of the University of Missouri (MU) herd of teaching horses. All MU herd horses were accommodated at either the MU Middlebush Farm (when on pasture) or the MU Veterinary Health Center VHC (during diet change). While some horses had underlying health conditions (Table S1), none were showing clinical signs at the time of sample collection or receiving any pharmacological treatments. All animal studies were approved by the MU IACUC (Protocol# 9037).

The experimental design consisted of six horses (referred to as the Diet Change [DC] group hereafter) which were removed from pasture at Middlebush Farm in mid-June to the MU VHC and switched to an all-hay diet upon entry to the VHC (Day −5). Following a five-day acclimation period, four of those six horses (Horses 1 through 4) then received a twice daily oral administration of metformin for seven days (from Day 1 through Day 7). Fecal samples were collected daily from Day −5 in the VHC through Day 7 from all six horses (12 samples/horse, with the exception of Horse 6 from which no Day 7 sample could be obtained), promptly transferred to a freezer, and maintained at −80 °C until DNA extraction could be performed. While at the VHC, horses were housed in individual stalls (4 m × 4 m) bedded with cedar shavings which were completely changed daily and provided approximately 10 pounds (4.5 kg) of Tall Fescue grass hay twice daily and ad libitum access to fresh drinking water. Daily exercise consisted of hand-walking within the VHC for approximately 10–15 min.

To control for random variability in the fecal microbiota over time, two paired fecal samples were collected one week apart from a separate cohort of five healthy (i.e., non-clinical) horses consistently accommodated at pasture, including 2 horses not included in the DC and metformin study, and three of the same horses included in that arm of the study (Table S1). Control samples were collected roughly 23 months after the DC period, and were handled and processed post-collection in the same manner as samples from DC horses (Figure S1).

All fecal samples from DC and control horses were then subjected to DNA extraction and 16S rRNA amplicon sequencing to characterize the change occurring following DC ± metformin, in contrast to the random change occurring in control horses.

Glucose tolerance was assessed via OST on Day 1 (prior to the initial dose of metformin) and Day 7 (after the final dose of metformin), in all horses in the DC group. Plasma ACTH, serum insulin, and blood glucose concentrations were also measured at those two time-points in those six horses.

### 2.2. Metformin Compounding and Administration

Metformin was compounded as a paste with a final concentration of 550 mg/mL, by a licensed pharmacist (Wedgewood Village Pharmacy LLC, Swedesboro, NJ, USA), and maintained at room temp during the study duration. Metformin was administered per os, twice daily, at 30 mg/kg body weight, to four of the six DC horses (Horses 1 through 4) after being switched from pasture to an all-hay diet.

### 2.3. Clinical Pathology Testing

All clinical pathology testing was performed at the MU VHC Clinical Pathology Laboratory, affiliated with the MU Veterinary Medical Diagnostic Laboratory. All blood samples were held on ice until centrifugation within two hours of collection, frozen, and analyzed as a single batch.

#### 2.3.1. Plasma ACTH Concentration

Plasma ACTH concentration was determined following centrifugation of ethylenediaminetetraacetic acid (EDTA)-anti-coagulated blood using a solid-phase, two-site sequential chemiluminescent immunometric assay (Immulite 2000 XPi, Siemens Healthineers, Erlangen, Germany).

#### 2.3.2. Blood Glucose Concentration

Blood glucose concentration was determined following centrifugation of heparinized blood by the hexokinase method (Beckman AU480 Chemistry Analyzer, Beckman Coulter, Brea, CA, USA).

#### 2.3.3. Serum Insulin Concentration

Serum insulin concentration was determined using a solid-phase, enzyme-labeled chemiluminescent immunometric assay (Immulite 2000 XPi).

#### 2.3.4. Oral Sugar Test

The OST protocol followed a published method [24]. Horses were fasted for 10 h prior to each OST. Briefly, blood was obtained via jugular venipuncture prior to and at +1 h and +2 h following oral administration of Karo™ Light Corn Syrup (ACH FOOD companies, Oakbrook Terrace, IL, USA) at 0.15 mL/kg body weight using a 60 mL dosing syringe. Blood was collected into Vacutainer™ vials (Becton Dickinson, Franklin Lakes, NJ, USA) that either did not contain an anti-coagulant (for measurement of serum insulin concentration) or contained EDTA (for measurement of plasma ACTH concentration) or sodium heparin (for measurement of plasma glucose concentration). Horses were allowed to resume consumption of hay after collection of the +2 h blood sample.

### 2.4. Fecal Sample Collection and DNA Extraction

All fecal samples were freely evacuated and collected off of the ground, being sure to remove exterior portions of the fecal bolus and collect interior portions for downstream processing and analysis. Following collection into sealed plastic vials, all fecal samples were transported to the laboratory on ice and maintained frozen until DNA analysis on all samples could be performed as a single batch. Fecal DNA was extracted using QIAamp PowerFecal kits (Qiagen, Germantown, MD, USA), according to the manufacturer’s instructions, with the following exception. Rather than disaggregation in the bead tubes using a vortex and adaptor, bead tubes containing sample and lysis buffer were homogenized at full speed (30 Hz) for three minutes using a TissueLyser II (Qiagen). DNA was eluted in 100 µL buffer and DNA concentrations were determined using Quant-iT dsDNA assay kits, broad range (Thermo Fisher, Waltham, MA, USA) and a Qubit 2.0 fluorometer (Thermo Fisher) and diluted to a standard volume and concentration.

### 2.5. 16S rRNA Amplicon Library Preparation and Sequencing

Extracted fecal DNA was processed at the MU DNA Core Facility. Bacterial 16S rRNA amplicons were constructed via amplification of the V4 region of the 16S rRNA gene with universal primers (U515F/806R) previously developed against the V4 region, flanked by Illumina standard adapter sequences [25,26]. Oligonucleotide sequences are available at proBase [27]. Dual-indexed forward and reverse primers were used in all reactions. PCR was performed in 50 µL reactions containing 100 ng metagenomic DNA, primers (0.2 µM each), dNTPs (200 µM each), and Phusion high-fidelity DNA polymerase (1U). Amplification parameters were 98 °C^(3min)^ + [98 °C^(15s)^ + 50 °C^(30s)^ + 72 °C^(30s)^] × 25 cycles + 72 °C^(7min)^. Amplicon pools (5 µL/reaction) were combined, thoroughly mixed, and then purified by addition of Axygen Axyprep MagPCR clean-up beads to an equal volume of 50 µL of amplicons and incubated for 15 min at room temperature. Products were then washed multiple times with 80% ethanol and the dried pellet was resuspended in 32.5 µL EB buffer, incubated for two minutes at room temperature, and placed on the magnetic stand for five minutes. The final amplicon pool was evaluated using the Advanced Analytical Fragment Analyzer automated electrophoresis system (Agilent, Santa Clara, CA, USA), quantified using quant-iT HS dsDNA reagent kits, and diluted according to Illumina’s standard protocol for sequencing on the MiSeq instrument.

### 2.6. Bioinformatics

The DNA sequences were assembled and annotated at the MU Informatics Research Core Facility. Primers were designed to match the 5′ ends of the forward and reverse reads. Cutadapt [28] (version 2.6; https://github.com/marcelm/cutadapt, accessed on 14 July 2019) was used to remove the primer from the 5′ end of the forward read. If found, the reverse complement of the primer to the reverse read was then removed from the forward read as were all bases downstream. Thus, a forward read could be trimmed at both ends if the insert was shorter than the amplicon length. The same approach was used on the reverse read, but with the primers in the opposite roles. Read pairs were rejected if one read or the other did not match a 5′ primer, and an error-rate of 0.1 was allowed. Two passes were made over each read to ensure removal of the second primer. A minimal overlap of 3 with the 3′ end of the primer sequence was required for removal.

The QIIME2 [29] DADA2 [30] plugin (version 1.10.0) was used to denoise, de-replicate, and count amplicon sequence variants (ASVs), incorporating the following parameters: (1) forward and reverse reads were truncated to 150 bases, (2) forward and reverse reads with number of expected errors higher than 2.0 were discarded, and (3) Chimeras were detected using the “consensus” method and removed. R version 3.5.1 and Biom version 2.1.7 were used in QIIME2. Taxonomies were assigned to final sequences using the Silva.v132 database [31], using the classify-sklearn procedure.

### 2.7. Data Availability

The 16S rRNA amplicon sequencing data described in the current study have been deposited in the NCBI Sequence Read Archive (SRA) and are available as BioProject PRJNA679208.

### 2.8. Statistics

All univariate data were first tested for normality and non-normal data were tested using non-parametric methods. Differences in insulin and glucose levels during OST were tested using a three-factor analysis of variance (ANOVA) with Horse, Day, and Time as factors. Differences in ACTH levels were tested using a two-factor ANOVA with Horse and Day as factors. Differences in intra-subject Jaccard similarity were performed using one-factor ANOVA with Dunn’s post hoc test. Significance was established as *p* < 0.05, the aforementioned testing was performed using SigmaPlot 14.0 (Systat Software, Inc., San Jose, CA, USA). Serial t-tests for differences between baseline and endpoint relative abundance of all ASVs were performed using MetaboAnalyst software [32], and raw *p*-values were corrected using the method of Benjamini and Hochberg [33] and a false discovery rate (FDR) of 10%.

Differences in multivariate data (e.g., β-diversity) were tested using permutational multivariate ANOVA (PERMANOVA) based on Jaccard or Bray–Curtis similarities, performed using Past3 software [34]. Past3 software was also used to generate the Jaccard distance matrices used to determine mean intra-subject similarities, and calculate Shannon and Simpson α-diversity indices using a rarefied dataset, subsampled to a uniform sequence count.

## 3. Results

### 3.1. Diet Change and Metformin Result in Normalization of Blood Glucose and Plasma Serum Insulin

**Figure 1:**
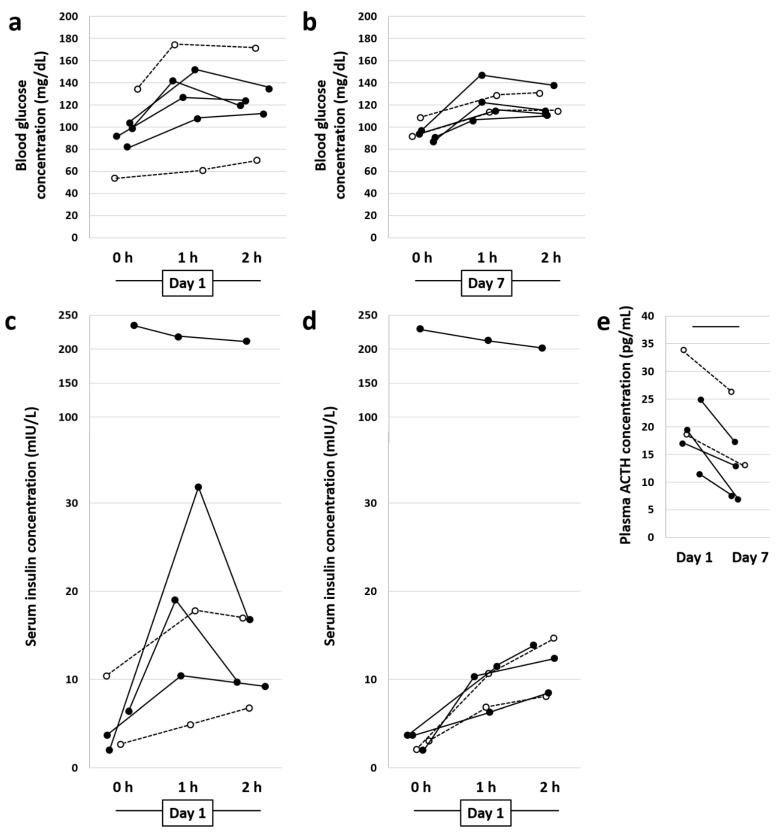
Results of bloodwork. Dot plots showing blood glucose levels (mg/dL) at 0 h (0 h), 1 h, and 2 h on Day 1 (**a**) and Day 7 (**b**) of the metformin treatment; serum insulin levels (mIU/L) during an oral sugar test at 0 h, 1 h, and 2 h on Day 1 (**c**) and Day 7 (**d**) of the metformin treatment; and plasma ACTH levels (pg/mL) on Day 1 and Day 7 of the study (**e**). In all panels, closed circles represent horses receiving metformin (*n* = 4) and open circles represent metformin controls that were also switched to an all-hay diet (*n* = 2). Bar indicates *p* < 0.05, paired t-test.

Evaluation of blood glucose revealed that, while all six horses were normoglycemic pre-treatment, there was considerable variability between horses (Figure 1a), and that variability was noticeably reduced at the post-treatment time-point (Figure 1b). Measurement of serum insulin levels indicated basal hyperinsulinemia in one of the six horses (Horse 2), interpreted as ID (Figure 1c). As with the blood glucose levels, while there was no day- or time-dependent difference detected using two-factor ANOVA, the variability between horses in serum insulin levels decreased post-treatment (Figure 1d). Results of OSTs were within normal limits with the exception of the one horse (Horse 2) in which ID was identified based on resting hyperinsulinemia. Additionally, plasma levels of ACTH were within the reference range in all six horses throughout the study duration but were significantly reduced on day 7 compared to pre-treatment (*p* < 0.05, Figure 1e), whether the horse with ID was included in the dataset or not. No apparent differences were observed between the four horses receiving metformin and the two control horses, although the fact that only two horses did not receive metformin precludes robust statistical analysis.

### 3.2. Diet Change and Metformin Result in Shift in β-Diversity and Proliferation of the Phylum Kiritimatiellaeota

**Figure 2:**
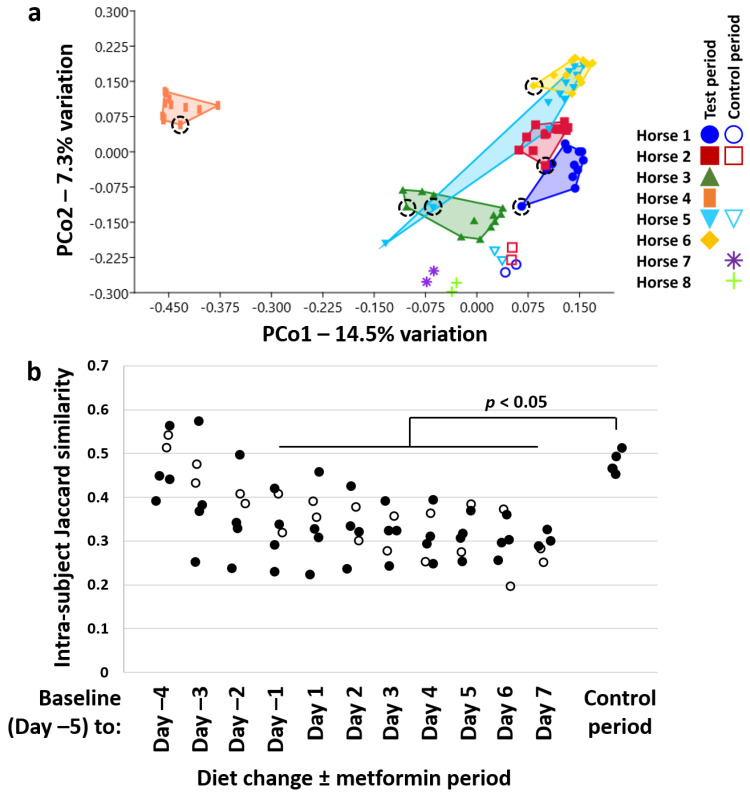
Consistent change in β-diversity rapidly following diet change. Principal coordinate analysis (PCoA) plot ordinated using Jaccard similarities, showing the relationship in β-diversity among fecal samples collected daily from the six horses (color-coded, legend at right) in the study, and paired samples from five control horses remaining on pasture, collected one week apart. Samples denoted by like symbols came from the same horse. Dotted circles indicate the initial sample from each horse upon movement off pasture (**a**); dot plot showing intra-subject Jaccard similarity between baseline (Day −5) and each successive day off pasture in the horses switched to an all-hay diet (*n* = 6) and receiving metformin (*n* = 4, closed circles), and between horses at Day 1 and Day 7 of the Control period (*n* = 5). Bracket indicates *p* < 0.05 versus control, ANOVA with Dunn’s post hoc (**b**).

Sequencing resulted in a mean (±SD) of 90,656 (±29,485) reads/sample, and all data were randomly subsampled to a uniform coverage of 37,934 reads/sample. For a comprehensive assessment of β-diversity over time, principal coordinate analysis (PCoA) and PERMANOVA were used. Figure 2a shows a PCoA plot comparing the six horses across time following the change from pasture to hay, alongside paired samples from five control horses, collected one week apart while the horses remained on pasture. While significant differences between DC horses were confirmed (*p* = 0.0001, *F* = 6.3), similar patterns were observed in the directionality of samples collected across time within each horse, with the initial sample from each DC horse consistently positioned on the left side of PCo1 or low on PCo2, with subsequent samples moving to the right of PCo1 and up along PCo2. Horses 3 and 6, which did not receive metformin, show a similar pattern of change to the other horses over time. Similar comparison using a weighted Bray–Curtis similarity revealed an almost identical pattern (Figure S2). Additionally, the paired control samples showed a greater degree of intra-subject similarity compared to samples collected at comparable time-points following DC ± administration of metformin. The gradual decrease over time in intra-subject similarity, shown in Figure 2b, reached statistical significance by Day −2, three days after removal from pasture. This significant (*p* < 0.05) reduction in intra-subject similarity by Day −2 was detected, regardless of whether the two horses not receiving metformin were included or removed from the analysis. While impossible to interpret conclusively, the change over time observed in the two DC horses not receiving metformin were comparable to those receiving metformin. Collectively, these data suggest that DC induced compositional changes in the GM among all six horses greater than would be expected to occur at random, while conclusions regarding the effect of metformin cannot be made. The conserved directionality of movement along PCo1 suggests that there is consistency to these diet/treatment-induced changes over time. Simpson and Shannon diversity were also assessed; however, no differences were detected over time in horses removed from pasture, or between the experimental and control periods, in either metric.

**Figure 3:**
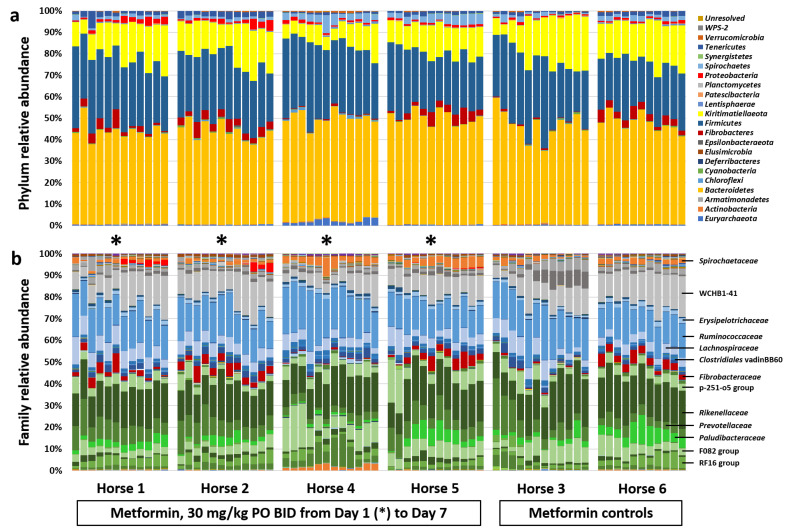
Changes in relative abundance of dominant taxa over time following diet change. Stacked bar chart showing the relative abundance of bacterial DNA detected in the feces of horses switched to an all-hay diet (*n* = 6), at the taxonomic level of phylum (**a**) and family (**b**). Samples for each horse are shown in chronological order, and the initiation of metformin treatment is denoted by an asterisk. Legend for dominant taxa shown at right.

**Figure 4:**
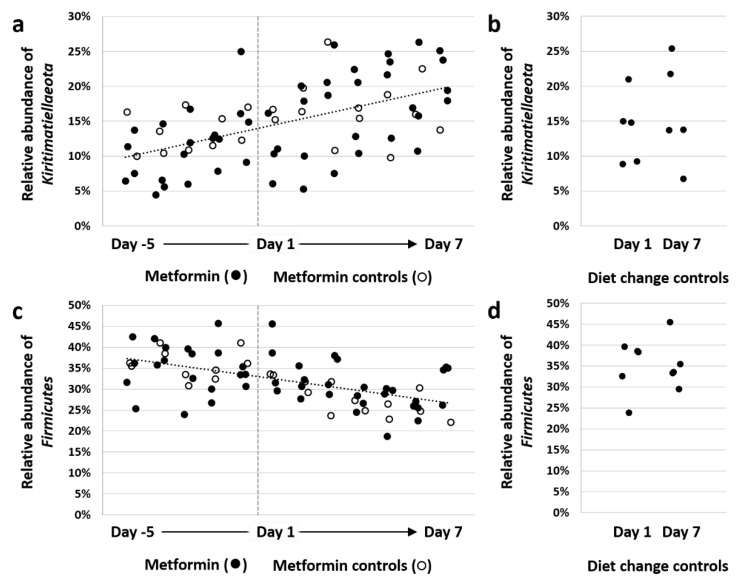
Change in Kiritimatiellaeota and Firmicutes over time following diet change. Dot plot showing the relative abundance of Kiritimatiellaeota in feces of horses switched to an all-hay diet on Day −5, and administered metformin (closed circles) from Day 1 through Day 7 (*n* = 6, **a**); and feces of horses maintained on pasture and not receiving metformin at Day 1 and Day 7 (*n* = 5, **b**); and dot plots showing the relative abundance of Firmicutes in those same experimental (**c**) and control (**d**) horses. Vertical grey line in panels (**a**,**c**) denotes the initiation of metformin administration in Horses 1 to 4.

Subjective assessment of the microbial community within each horse at the taxonomic level of phylum revealed the dominance of Bacteroidetes, Firmicutes, and Kiritimatiellaeota, in decreasing order of mean relative abundance, with appreciable levels of Fibrobacteres, Proteobacteria, Spirochaetes, and Tenericutes as well (Figure 3a). Two-factor ANOVA, used to test for differences in the relative abundance of each phylum, detected a time-dependent increase in *Kiritimatiellaeota* (*p* < 0.001, *F* = 4.3), and decrease in Firmicutes (*p* = 0.003, *F* = 3.1). Resolved to the level of family, Kiritimatiellaeota comprised three primary clades all within order WCHB1-41 (Figure 3b), encompassing a total of >670 distinct amplicon sequence variants (ASVs) (Figure S3). The percentage of total DNA in horses switching to an all-hay diet, regardless of the presence or absence of metformin, demonstrated a steady increase in Kiritimatiellaeota, reaching significance by Day −1 (*p* < 0.05, ANOVA) (Figure 4a). No difference was detected in the relative abundance of Kiritimatiellaeota between control horses at Day 1 and Day 7 (Figure 4b), or between control horses at Day 1 and DC horses at Day 1, collectively indicating a steady increase in Kiritimatiellaeota in horses switching from pasture to an all-hay diet. The opposite effects were observed in the relative abundance of Firmicutes, with a steady decrease in DC horses over time (Figure 4c), and no difference over time in the control horses (Figure 4d).

To identify all taxa associated with these changes, serial t-tests were performed comparing the relative abundance of all ASVs in baseline and endpoint samples from the six horses switched to an all-hay diet. While 107 of the 3902 ASVs tested yielded raw *p*-values below 0.05, only three ASVs withstood a conservative correction for multiple tests. ASVs annotated to order WCHB1-41 of the poorly resolved phylum Kiritimatiellaeota, and the family Ruminococcaceae (best match: *Phocea* sp., 92.06% nucleotide identity) were significantly increased in abundance after the change in diet and metformin administration, while an ASV annotated as Lachnospiraceae XPB1014 group (best match: *Ruminococcus gnavus*, 94.09% nucleotide identity) was significantly reduced. A full list of the 107 ASVs with raw *p*-values below 0.05 is provided in Table S2. Several taxonomies appear repeatedly, and with consistent directions of change, among them Kiritimatiellaeota (16 ASVs with raw *p* < 0.05, and 15 of 16 enriched at endpoint relative to baseline) and Rikenellaceae RC9 gut group (12 ASVs with raw *p* < 0.05, and all 12 enriched at endpoint relative to baseline). In contrast, other common annotations with raw *p* < 0.05 such as Ruminococcaceae UCG-010 and NK4A214 group were variable, with different identically annotated ASVs showing both directions of change following treatment. Notably, a retrospective search of the Ribosomal Database Project (RDP) for the best match against ASVs annotated to the phylum Kiritimatiellaeota, consistently resulted in matches less than 85% nucleotide identity to any 16S rRNA sequences in the RDP, indicating that these bacteria represent poorly characterized, previously uncultured taxa.

## 4. Discussion

Collectively, these data provide compelling evidence for consistent diet-induced changes in the fecal microbiota of healthy horses, characterized by an expansion of the phylum Kiritimatiellaeota and concomitant reduction in *Firmicutes*. While the low number of horses removed from pasture but not receiving metformin (*n* = 2) precludes meaningful statistical analysis, it is difficult to ascribe substantial effect to metformin administration per se, as the observed changes appear to begin in all six horses upon removal from pasture. Kiritimatiellaeota are a poorly characterized phylum, proposed in 2016 as a novel phylum distinct from the Planctomycetes, Verrucomicrobia, Chlamydiae (PVC) superphylum [35]. This monophyletic clade, previously recognized as Verrucomicrobia subdivision 5 [36], was poorly resolved using our 16S rRNA primers, and future work will need to explore other target regions or molecular platforms to better characterize these commensal taxa. A recent report of four draft single cell genomes of Kiritimatiellaeota from the continental deep subsurface, alongside previous reports in the equine gut [37], highlights that these are ubiquitous environmental taxa as well. The first reported cultivation of members of Kiritimatiellaeota interpreted the phenotypic and genomic data to suggest that related bacteria are specifically adapted to, “the utilization of sulfated glycopolymers produced in microbial mats or biofilms” [35]. This is notable due to their phylogenetic proximity to the Verrucomicrobia, the type species being *Akkermansia muciniphila*, a known mucin degrader that resides in the heavily sulfonated mucin layers of the lower GI tract of most mammalian hosts. Indeed, sequences were detected in the current data which were annotated to the phylum Verrucomicrobia; however, these constituted a very small portion of the fecal microbiota and showed no change in relative abundance over time in the DC horses. Thus, while horses appear to possess true Verrucomicrobia in their hindgut, our data suggest that Kiritimatiellaeota is a dominant mucosal colonizer of horses.

It is worth noting that many previously published reports of the equine hindgut include *Verrucomicrobia* as a dominant phylum. This speaks to the necessity of constant database curation and the use of the most current database and software by investigators. As an example of the latter, the current dataset, when analyzed using QIIME1.9 [38], annotated the Kiritimatiellaeota sequences as group RFP12 within the Verrucomicrobia subgroup 5, while the newer and more accurate annotations provided by QIIME2 [29,39] indicate that these sequences are more accurately identified as Kiritimatiellaeota.

While we are unaware of any studies describing the effect of metformin on the equine microbiota, early work from Willing et al. using molecular methods and a robust cross-over design identified several significant diet-dependent differences in the composition of the equine fecal microbiota [40], although all grass diets were not included. Daly et al. demonstrated that horses consuming grass or concentrate-based diets had different hindgut communities with concentrate-consuming horses harboring greater numbers of certain Lachnospiraceae, Bacteroidetes, and lactic acid-producing bacteria, which was validated by higher concentrations of lactic acid in the cecal contents [41]. Shortly thereafter, Fernandes et al. confirmed that an abrupt change in diet was associated with a change in the fecal microbiota, although in that study, horses were transitioned from forage to pasture [42]. The most relevant publication is the recent report from Garber et al., describing a crossover study wherein six adult Welsh ponies were abruptly switched from grass to hay, and from hay to grass. In that study, an all-hay diet was associated with a reduced relative abundance of Verrucomicrobia, which rebounded following return to grass, and Kiritimatiellaeota was not discussed. Unfortunately, geographical and breed-dependent differences notwithstanding, those data were also analyzed using the older QIIME1.9, making inter-study comparison of annotations difficult.

Salem et al. showed that the fecal microbiota of horses on pasture undergoes a considerable amount of variation over the course of a year [43]. While the samples used for controls in the present study were collected a year after the DC/metformin study was performed, they were collected at roughly the same time of year, from horses at the same farm (including some of the same horses) as those used in the DC/metformin study. While the study by Salem et al. does convincingly show changes in the fecal microbiota of their study population over time, close examination of the data also shows that, in the absence of management changes (e.g., supplementation of pasture diet with haylage), the change in any given horse between successive time-points is much less, as was observed in our study.

One study comparing horses with EMS and healthy control horses [17] found significantly reduced α-diversity in fecal samples from horses with EMS. Notably, an unclassified taxon within Verrucomicrobia subdivision 5 yielded the highest linear discriminant analysis (LDA) score in an LDA effect size (LEfSe) analysis, suggesting this taxon was preferentially associated with EMS. This introduces the question of whether these represented true Verrucomicrobia or Kiritimatiellaeota, and if the latter, how this can be reconciled with an increase in its relative abundance during DC and treatment with metformin. One explanation, while speculative, is that the effects of diet change and metformin on the GM might differ between health and affected horses. While the single horse in our study diagnosed as having ID based on basal hyperinsulinemia did not harbor appreciably greater relative abundance of Verrucomicrobia or Kiritimatiellaeota than other horses in the study, it is only a single horse.

The horse in question was subclinical and thus retained in the study to avoid losing one subject in an already modestly powered study. It should be noted however, that the same increase in Kiritimatiellaeota and Rikenellaceae observed in the other five horses were found in the horse with ID as well, and that horse was clustered closely with other experimental horses deemed healthy upon physical examination. Thus, in this individual horse, no obvious differentiating features were detected in the sequencing data.

Thus, the primary limitations of the current study are the modest sample size in the DC group, a direct result of the expense and logistical considerations associated with those studies, and the fact that horses were ostensibly healthy. While these studies provide important information regarding the effects of transition to an all-hay diet in healthy horses, we were unable to perform a controlled study of this nature in multiple horses affected with EMS, and the one horse with ID that was included in the DC group was not showing clinical signs often associated with EMS (e.g., laminitis, obesity) at the time of sample collection. Future studies will rely on banked fecal samples accrued from patients presenting at the VHC.

## 5. Conclusions

Collectively, these data demonstrate a shift in composition of the equine fecal microbiota following a switch from pasture to an all-hay diet, characterized by an increase in the poorly characterized Kiritimatiellaeota and decrease in various members of the phylum *Firmicutes*. Future studies are needed to test the effects of these treatments on larger groups of horses with EMS. Our findings also underscore the presence of Verrucomicrobia and Kiritimatiellaeota as two distinct taxonomies in the equine hindgut, possibly with distinct influences on host physiology. Additional work is needed to isolate and better characterize these dominant taxa in the equine hindgut, as they may serve as useful fecal biomarkers or therapeutic targets.

## Data Availability

The 16S rRNA amplicon sequencing data described in the current study have been deposited in the NCBI Sequence Read Archive (SRA) and are available as BioProject PRJNA679208.

## Supplementary Materials

The following are available online at https://www.mdpi.com/article/10.3390/ani11040976/s1, Figure S1: Experimental design, Figure S2: Consistent change in weighted β-diversity rapidly following diet change, Figure S3: Change in ASVs within Kiritimatiellaeota over time following diet change, Table S1: Demographics, Table S2: ASVs differing between baseline and endpoint.
