# Supplementary figures and images for: The Influence of Diet Change and Oral Metformin on Blood Glucose Regulation and the Fecal Microbiota of Healthy Horses
Source: Animals (Basel). 2021 Apr 1;11(4):976. doi: 10.3390/ani11040976 (PMC8065426; doi:10.3390/ani11040976)

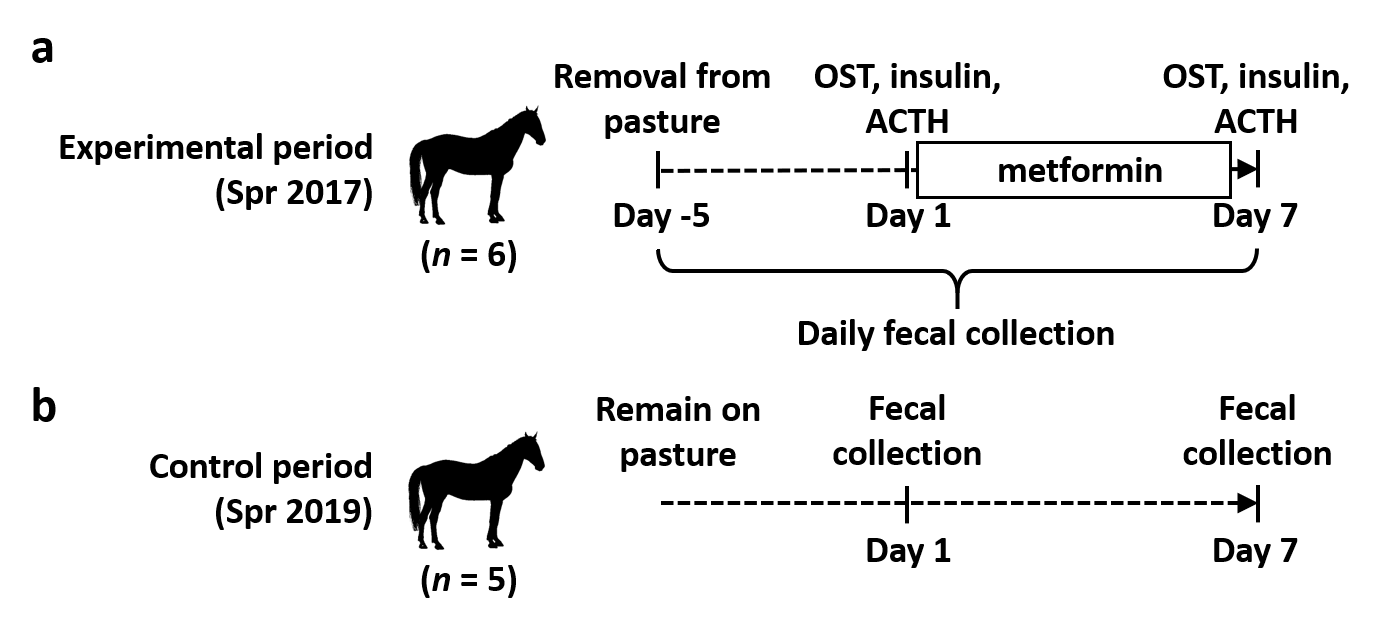

Supplement: Supplementary file 1 [file animals-11-00976-s001.zip › Suppl_Fig1.tif]

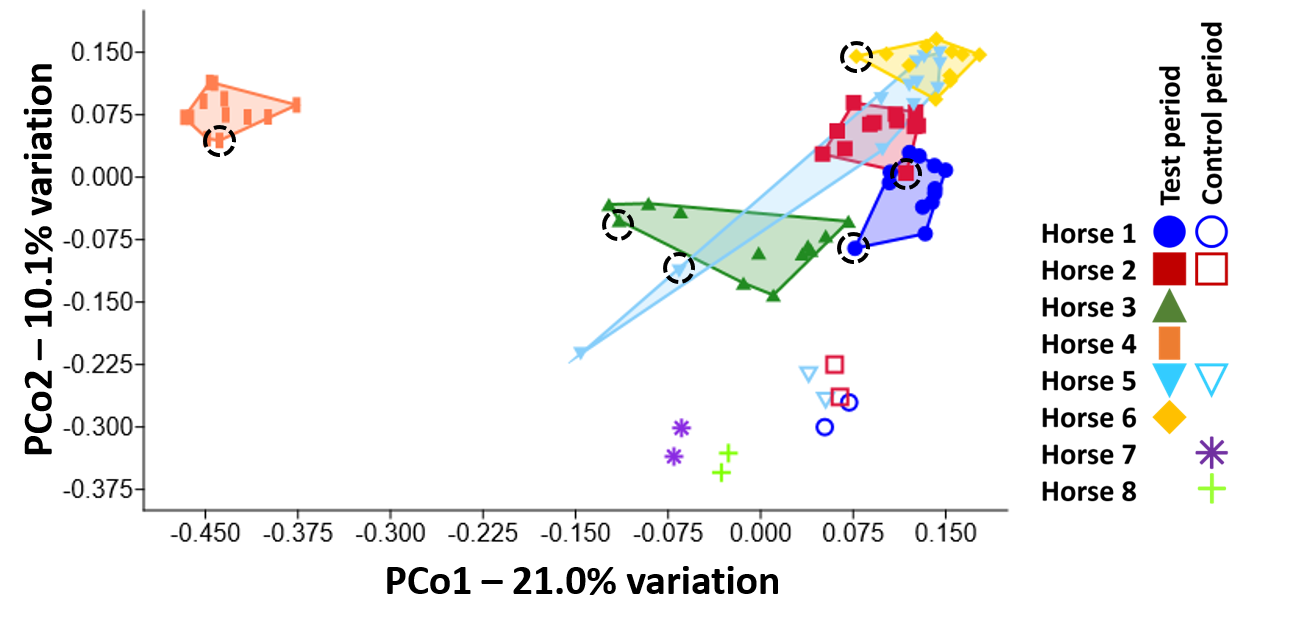

Supplement: Supplementary file 1 [file animals-11-00976-s001.zip › Suppl_Fig2.tif]

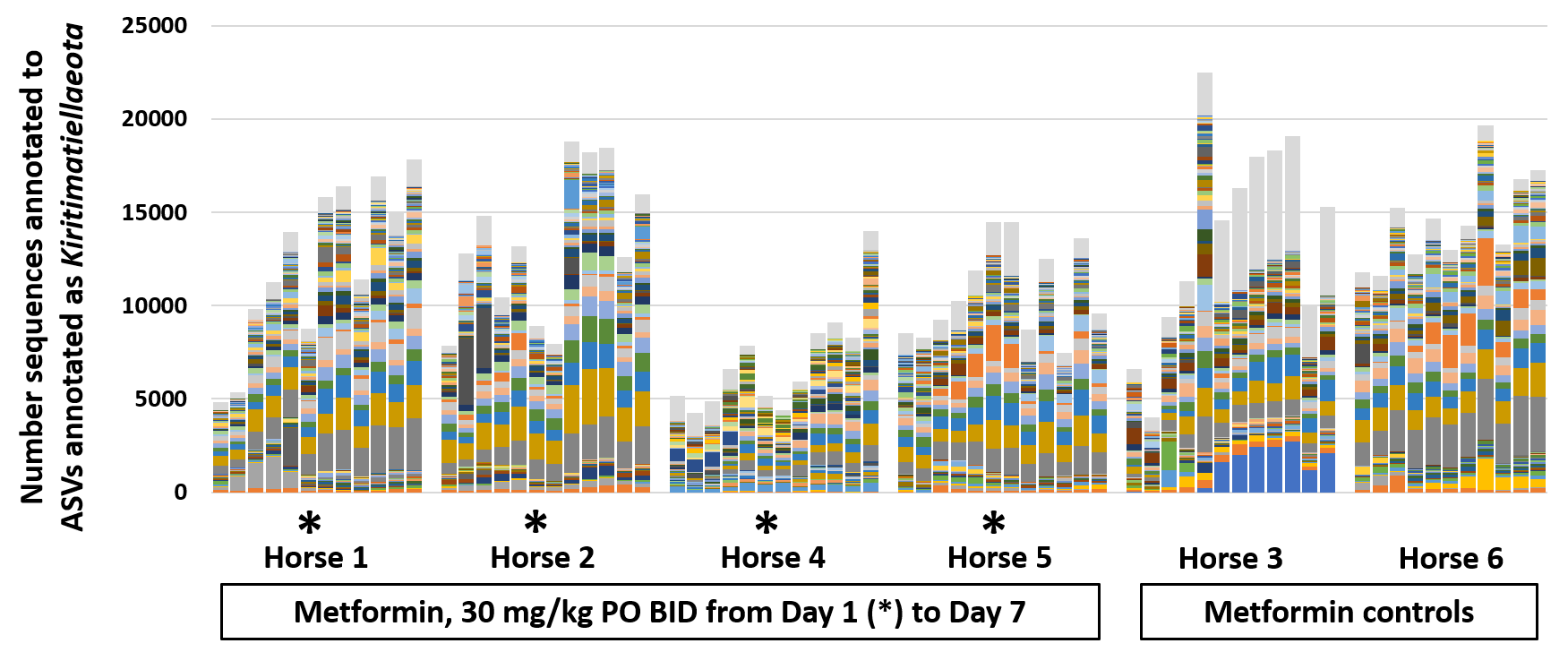

Supplement: Supplementary file 1 [file animals-11-00976-s001.zip › Suppl_Fig3.tif]
